# Editorial: Global excellence in brain disease mechanisms: Asia & Australasia

**DOI:** 10.3389/fnmol.2023.1279769

**Published:** 2023-08-29

**Authors:** Grace Cunliffe, Xin Yi Yeo, Sangyong Jung

**Affiliations:** ^1^Institute of Molecular and Cell Biology (IMCB), Agency for Science, Technology and Research (A^*^STAR), Singapore, Singapore; ^2^Division of Neuroscience and Experimental Psychology, School of Biological Sciences, Faculty of Biology, Medicine and Health, University of Manchester, Manchester, United Kingdom; ^3^Department of Psychological Medicine, Yong Loo Lin School of Medicine, National University of Singapore, Singapore, Singapore; ^4^Department of Medical Science, College of Medicine, CHA University, Seongnam, Republic of Korea

**Keywords:** neurodevelopment, neurodegenerative, genetic mutations, synaptic alterations, brain disease, neurological function, environmental factors

The establishment of the basic architecture of the brain requires the formation of connections between neuronal axons and dendrites, which results in the generation of functional neural circuits (Pang et al., 2023). These processes are influenced by a combination of genetic and environmental factors unique to each individual (Karmiloff-Smith et al., 2014). Neurodevelopmental disorders such as autism spectrum disorder are associated with the dysregulation of developmental processes involved in the initial formation of neuronal physical structures and synaptic connections (Prem et al., 2020), whilst alterations in the maintenance of mature neuron and network function drive the development of later-life neuropsychiatric and neurodegenerative conditions (Jellinger, 2010; Winkelmann et al., 2014).

As a result of the complex and multifactorial nature of determinants of neurological structure and function, exact causes of neurocognitive, neurodevelopmental, and neurodegenerative conditions are notoriously difficult to pinpoint. Current treatments for brain conditions are therefore largely inadequate. Continuous progress in neuroscience is essential to provide new perspectives on the contribution of interactions between gene variations, normal cellular function, and environmental changes toward neurological disorders, allowing the translatability of new concepts into therapeutics to be more effectively considered. As such, this Research Topic gathers recent advancements in neuroscience research, focusing on work conducted in Asia and Australasia, that attempts to link genetic and environmental factors to brain disease mechanisms.

Here, through whole-exosome sequencing, Chen et al. reveal four novel p53-related protein kinase (TP53RK) gene variants associated with Galloway-Mowat syndrome (GAMOS) in Chinese children. GAMOS is a genetically heterogeneous, autosomal recessive disorder characterized by brain developmental impairment and presenting with microcephaly, developmental delay, and intellectual disability. Pathology in GAMOS type 4 (GAMOS4) results from defective TP53RK function. Interestingly, nephrotic syndromes are absent from patients in this study, in contrast to observations in previously reported GAMOS4 cases. This work improves mechanistic and diagnostic insights into the disease, highlights the potential impact of single gene mutations on neurodevelopment, and suggests the contribution of alternate factors to disease manifestation, reminiscent of the majority of neurodevelopment disorders.

Lee et al. describe how the presence of known mutations in the chromatin remodeler *CHD8* gene may contribute toward brain alterations underlying autism spectrum disorder (ASD) presentation. Interestingly, this paper highlights the sex- and age-dependency of CHD8 mutations on synaptic and transcriptomic changes in mice. Specifically, the authors report that CHD8 mutations result in reductions in excitatory synaptic transmission in juvenile male mice, that correlated with ASD-like behaviors. However, mutations lead to increased excitatory transmission in female mice with comparatively normal behavior, suggesting that reductions in the neuronal excitatory-inhibitory balance may account for ASD development in a sex-dependent manner.

Gene variants are also widely recognized to contribute to aging-associated neurodegenerative disease development. In their review, Wei et al. describe how Parkinson's disease (PD) pathology may arise from mutations in the leucine-rich repeat kinase 2 (LRRK2) coding gene—one of the most common gene modifications in human PD patients (Kingwell, 2023). LRRK2 functions as a central hub modulating the activity of Golgi proteins, which in turn regulate the integrity and trafficking of intracellular vesicles and the trans-Golgi network organelle function (Piccoli and Volta, 2021). Aberrant LRRK2 functions are linked to the fragmentation of the Golgi Apparatus, leading to a build-up of toxic substances, aggregation of α-synuclein, and consequent oxidative stress and cell death characteristic of PD.

Alterations to vesicle transport and synaptic trafficking lead to synaptic loss and plasticity changes linked to prodromal neurodegeneration (Soukup et al., 2018; Pelucchi et al., 2022). In their study, Xu et al. investigate how the mutant huntingtin protein drives neurodegeneration in Huntington's disease (HD). They provide first-hand evidence of the involvement of altered inhibitory synaptic vesicle exocytosis, reduced bouton density and impairment of inhibitory neurotransmission at the presynaptic domains of striatal neurons in neurological pathology observed in the zQ175 rodent HD model. The study emphasizes the importance of cellular function maintenance for optimal neurological health.

Epigenetic modifications describe regulatory processes that control gene expression. Therefore, gene alterations depend not only on initial genetic variability in the DNA sequence but also on the nature of posttranscriptional RNA modifications. Fan et al. review specifically how N6-methyladenosine (m6A) methylation, the most abundant internal RNA modification in eukaryotic cells, is strongly linked to learning and memory processes in the brain. Although the precise role of m6A RNA methylation is unclear, changes in m6A levels in the nervous system are associated with synaptic alteration in aging and neurodegenerative diseases. This review presents a link between m6A methylation and the function of the neurovascular unit, and its likely neuroprotective effects which may contribute to neurofunction recovery in neurodegenerative disease.

Besides genetic alterations, the impact of environmental factors on neurological function cannot be understated. Shin and Lee investigated the effect of environmental-dependent, early-life stress associated with maternal separation on cognitive development in mouse pups. They observed that adolescent mice display varying alterations in cognition, dependent on their environmental context during exposure to neonatal maternal separation. This study therefore presents comprehensive early-life stress models that may provide the impetus for further understanding environmental contributions to neuropsychiatric conditions such as anxiety and depression.

In addition to their common standalone occurrence, psychiatric conditions often present as comorbidities of a range of neurological disorders. However, current treatment options are known to result in further neurological disturbance and new psychiatric side effects (Haddad and Dursun, 2008). Therefore, increasing the understanding of specific brain circuitry changes that underlie neuropsychiatric disorders is of significant research interest. In their commentary, Zhao et al. point to the role of ventral tegmental area projections to the basolateral amygdala in encoding anxiety, but not depressive-like behaviors, presenting a novel neural circuitry target for anxiety-specific treatment. This study emphasizes the importance of studying anxiety and depression as independent disorders involving unique brain circuitry. A deeper understanding of these distinct changes can promote the development of more effective, highly-targeted treatments with improved therapeutic outcomes and fewer side effects.

The studies presented in this Research Topic highlight novel insights into the involvement of genetic and environmental factors in neurological function and pathology. We hope that these findings from experts within the field will contribute to enhancing our understanding and thus promoting further advancements in the prevention, diagnosis, and treatment of brain diseases.

## Author contributions

GC: Writing—original draft. XY: Writing—review and editing. SJ: Writing—review and editing, Writing—original draft.
